# Discovering Topic-Oriented Highly Interactive Online Communities

**DOI:** 10.3389/fdata.2019.00010

**Published:** 2019-06-06

**Authors:** Swarna Das, Md Musfique Anwar

**Affiliations:** Department of Computer Science and Engineering Jahangirnagar University, Dhaka, Bangladesh

**Keywords:** online social network, interaction strength, active community, query cohesiveness, structure cohesiveness

## Abstract

Community detection is an interesting field of online social networks. Most existing approaches either consider common attributes of social network users or rely on only social connections among the users. However, not enough attention is paid to the degree of interactions among the community members in the retrieved communities, resulting in less interactive community members. This inactivity will create problems for many businesses as they require highly interactive users to efficiently advertise their marketing information. In this paper, we propose a model to detect topic-oriented densely-connected communities in which community members have active interactions among each other. We conduct experiments on a real dataset to demonstrate the effectiveness of our proposed approach.

## 1. Introduction

Nowadays, Online Social Networks (OSN) are widely used by a large part of the general population. Similar interests, choices, and hobbies tend to form a group of users in a social network known as online community. There have been many attempts to detect these online communities for the purpose of business, marketing, recommendations, biological research, etc. Often the mere use of connection links does not provide an effective group of users. As a result, these groups do not bring efficient results.

There are two types of network topology. One is global, where information of a whole network is captured and another is local, i.e., a network that works with the similar nodes (Tang et al., 2017). There have been many approaches to detect communities and serve various other fields with it (Fortunato and Hric, 2016). An approach to detecting communities is Affinity Propagation, where the network is divided and a multiobjective evolutionary algorithm is introduced (Shang et al., 2016). For the purpose of local community formation, dynamic membership function can be used (Luo et al., 2018). Fuzzy relations can be used for non-overlapping community detection. The nearest node with each node's greater centrality and fuzzy relations are combined for the desired result (Luo et al., 2017).

Recent research works consider social users' topical interests in OSNs, e.g., (Yang et al., 2013), in order to find meaningful communities. However, these methods did not focus on the topical interactions among the community members. Therefore, such communities contain many members who have very inactive topical interactions among them which perform poorly in viral marketing. In order to avoid the inactivity problem authors (Lim and Datta, 2016) have proposed an approach where interaction pattern and frequency are considered rather than only counting the following/follower links.

Our observation is that social users have different degrees of topical intimacy among them. In this work, we propose an approach to discover topic-oriented *highly interactive* communities in OSNs, where the members in the community should have a certain degree of topical interactions with each other related to a given query. We also emphasize that the members in the retrieved communities should actively interact with at least *k* other members within the community. Below, we summarize our contributions:

- We propose a methodology to discover highly interactive online communities where community members have a high degree of interactions with each other on similar topics;
- We quantify the topical interaction strengths among the users;
- We perform experiments on a real dataset to demonstrate the effectiveness of the proposed method.

## 2. Related Work

Earlier methods for community detection are based on structural information of the social graph such as modularity (Clauset et al., 2004), edge betweenness (Newman and Park, 2003), and neighborhood concepts (Cohen, 2008). Some approaches also considered the textual content published by the users along with social connections to detect like-minded users. For example, SA-Cluster applied random-walk to measure the closeness of a node in an augmented attributed graph (Zhou et al., 2009). A Topic-Link LDA model (Liu et al., 2009) is proposed which considers both the linkage structure and similarities of the contents of edges to detect communities. A probabilistic generative model named as CESNA is proposed by Yang et al. (2013) and combines community memberships, node attributes, and the network topology to find the communities.

More recently, some approaches have focused on the interaction strength between the users in order to find active communities. Dev et al. (2014) considered the impact of interaction between users as well as the impact of the group behavior without considering topical attributes of the nodes. Lim and Datta (2016) proposed the Highly Interactive Community Detection (HICD) method, which constructs a weighted network using the frequency of direct interactions between users. Correa et al. (2012) proposed the *iTop* algorithm, which constructs a weighted graph based on user interactions and maximizes the local modularity to detect topic-oriented communities based on a set of seed users. However, all these methods ignored topic-wise users' inter-activeness. Our goal is to discover communities where users have high interactions with others with regard to the given query consisting a set of topics.

## 3. Methodology

First we formally formulate the problem of discovering highly interactive topical communities in OSNs. Then we give an overview of our proposed approach.

**Attributed Social Graph:** An attributed social graph is denoted as *G* = *(U, E*, A*)*, where *U* represents the set of social users (nodes), *E* indicates the set of links (edges) between the users, and A={*T*_1_, *T*_2_, …, *T*_*m*_} is the set of topics discussed by the social users in *G*.

In Twitter, users mention each other using “@.” In order to construct a link (*a, b*) between users, @mention is used, i.e., *M*_*a, b*_ denotes that user *a* has posted a tweet which contains @*b*.

***k*-Core:** Given an integer *k* (*k*≥ 0), the *k*-core of a graph *G*, denoted by *C*^*k*^, is the maximal connected sub graph of *G*, such that ∀*u* ∈ *C*^*k*^, $deg_{C^{k}}\left(u\right)$ ≥ *k*, where $deg_{C^{k}}\left(u\right)$ refers to the degree of a node *u* in *C*^*k*^. A *k*-core component $H_{j}^{k}$ is considered as a community from a structural point of view.

**Node Core Number:** The core number of a social user *u* in a *k*-core induced sub graph from *G* indicates the maximum *k* for which *u* belongs to that *k*-core sub graph.

**Topic:** A topic contains a set of related words that represents the topic. For example, the politics topic has words like election, vote, democracy, political party, etc.

**Activity:** Any action performed by a social user is referred to as an activity. For example, posting a new tweet or retweeting an existing tweet is considered as an activity. In our work, we consider only those actions that are performed between any two social users. For example, a user *u* in Twitter replies to a tweet posted by user *v*. This activity is recorded as an activity tuple 〈*u, v*, ψ_*uv*_〉, where ψ_*uv*_ indicates the set of attributes (topics) exchanged between *u* and *v* (Anwar et al., 2018).

**Query:** An input query *Q*={*T*_1_, *T*_2_…, *T*_*n*_} contains a set of query topics.

**Active Interaction Edge:** If any two social users *u* and *v* in *G* have a certain number of direct interactions (γ(≥ 1)) between them related to *Q*, then we consider the interaction link between those two users as an active interaction edge (*e*_*uv*_). Factor *w*_*uv*_ indicates their involvement in direct interactions compared with the most active pair of users in the network.

(1) $$\begin{array}{l} w_{uv}=\frac{\left|ACTS\left(u,v,\psi_{uv}\right)\right|}{max_{x,y\in U^{Q}}|ACTS\left(x,y,\psi_{xy}\right)|} \end{array}$$

where *ACTS*(*u, v*, ψ_*uv*_) indicates the number of direct interactions between *u* and *v* containing ψ_*uv*_ ⊆ *Q*.

**Active User:** The users of an active interaction edge *e*_*uv*_ are considered as active users. The set of all the active users for a given query *Q* is denoted as *U*^*Q*^.

### 3.1. Problem Definition

Given a graph *G* = *(U, E*, A*)*, an input query *Q* and an integer *k*, we first find the set of active edges between the social users by measuring interaction strength *w*_*uv*_(*w*_*uv*_ ∈ [0, 1]). Then an induced sub graph $H_{j}^{k}$ is considered as an active interactive community if it satisfies the following criteria.

1. ***Connectivity*. **$H_{j}^{k}\subset G$ is connected;
2. ***Structure cohesiveness*. **$\forall u\in H_{j}^{k}$ has interaction degree of at least *k*;
3. ***Active interaction*. **$\forall e_{uv}\in H_{j}^{k}$, the interaction strength of *e*_*uv*_ is *w*_*uv*_≥θ and θ ∈ [0, 1] is a threshold.

**Figure 1:**
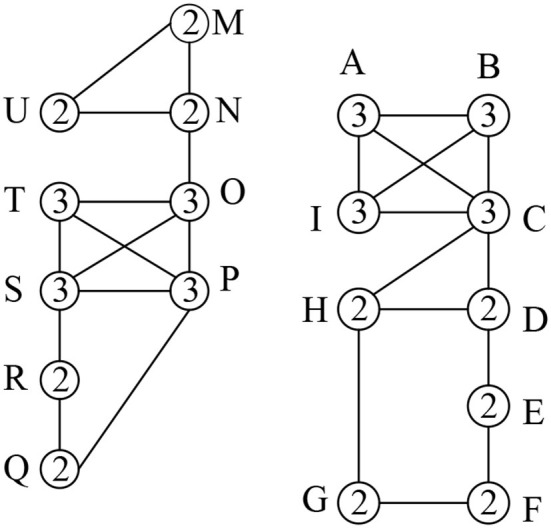
Social Graph (the number denotes the node core number).

Figure 1 shows a social graph *G* with the core number for each node, e.g., the three-core nodes are {A,B,C,I}. Table 1 represents the interaction frequencies among the users for topic *T*_1_ and *T*_2_. In Table 2, we show the interactive communities for a query *Q* = {*T*_1_, *T*_2_}. We get different community members for different values of *Q*, *k*, and θ. For example, when *Q*={*T*_1_}, *k* = {2}, and θ = {0.4}, we get $H_{1}^{2}$ = {A,B,C,I}, $H_{2}^{2}$ = {O,P,Q,R,S,T} while for the same values of *Q* and θ with an increase value of *k* = {3}, we get $H_{1}^{2}$ = {A,B,C,I}, $H_{2}^{2}$ = {O,P,S,T}. Again, for *Q*={*T*_1_, *T*_2_}, *k* = {2} and θ = {0.5}, we get $H_{1}^{2}$ = {A,B,C,D,H,I}, $H_{2}^{2}$ = {O,P,Q,R,S,T} and $H_{3}^{2}$ = {M,N,U}

**Table 1:** Interaction log.

**(e_*ab*_)**	***T*_1_**	***T*_2_**	**(e_*ab*_)**	***T*_1_**	***T*_2_**
(M,N)	6	18	(A,B)	20	7
(M,U)	3	14	(A,C)	18	6
(N,U)	5	15	(A,I)	14	6
(N,O)	6	8	(B,C)	10	13
(O,T)	20	13	(B,I)	13	8
(O,P)	19	7	(C,I)	15	9
(O,S)	11	6	(C,D)	7	12
(P,T)	14	8	(C,H)	6	17
(P,S)	18	9	(D,H)	12	9
(S,T)	16	9	(D,E)	7	18
(S,R)	12	4	(H,G)	5	8
(R,Q)	10	5	(E,F)	4	9
(P,Q)	20	9	(F,G)	6	20

**Table 2:** Community members for different values of *Q*, *k*, and θ.

**Query**	**Community**
*Q* = {*T*_1_}, *k* = {2}, θ={0.4}	$H_{1}^{2}$ = {A,B,C,I}, $H_{2}^{2}$ = {O,P,Q,R,S,T}
*Q* = {*T*_1_}, *k* = {3}, θ={0.4}	$H_{1}^{3}$ = {A,B,C,I},$H_{2}^{3}$ = {O,P,S,T}
*Q* = {*T*_2_}, *k* = {2}, θ={0.4}	$H_{1}^{2}$ = {B,C,D,E,F,G,H,I},$H_{2}^{2}$ = {M,N,O,P,S,T,U}
*Q* = {*T*_2_}, *k* = {2}, θ={0.5}	$H_{1}^{2}$ = {C,D,H},$H_{2}^{2}$ = {M,N,U}
*Q* = {*T*_1_,*T*_2_}, *k* = {2}, θ={0.4}	$H_{1}^{2}$ = {A,B,C,D,E,F,G,H,I},$H_{2}^{2}$ = {M,N,O,P,Q,R,S,T,U}
*Q* = {*T*_1_,*T*_2_}, *k* = {2}, θ={0.5}	$H_{1}^{2}$ = {A,B,C,D,H,I},$H_{2}^{2}$ = {O,P,Q,R,S,T},$H_{3}^{2}$ = {M,N,U}
*Q* = {*T*_1_,*T*_2_}, *k* = {3}, θ={0.4}	$H_{1}^{3}$ = {A,B,C,I},$H_{2}^{3}$ = {O,P,S,T}

### 3.2. Highly Interactive Community Detection Approach

In this work, we propose a method to detect highly interactive communities for a given a query *Q* in an online social attributed graph *G*. The desired communities from the graph *G* can be identified in the following three steps:

1. Identify the set of active users based on their direct interaction with each other for a given query *Q*.
2. Refine the original social graph *G* by filtering the inactive social users.
3. Apply *k*-core technique on the refined social graph in order to detect the desired online communities.

The first step of our approach is measuring the interaction frequencies among the users for a given query *Q* in social graph *G* to filter the weakly connected topology links. For this purpose, we consider users who have direct communication with others via retweets or mentions and consider an interaction link between two users irrespective of whether they have a topology link or not.

After establishing the newly active interaction edges and filtering the inactive topology links from the social graph *G*, we apply *k*-core on the refined social graph to find the connected components in which every node has degree of at-least *k*.

We develop an algorithmic framework to detect highly interactive communities for a given *Q*.

**Algorithm overview**. The algorithm, called Query Algorithm, has three steps. First, it computes the interaction strength *w*_*uv*_ of each edge *e*_*uv*_ for a given query *Q* in order to find the set of active users (line 1-5). Next, we compute the induced sub graph *G*^*Q*^ from *U*^*Q*^ (line 6). Finally, we identify the maximal *k*-core of *C*^*k*^(*G*^*Q*^) from the induced graph *G*^*Q*^ to find the set of active connected components (i.e., desired connected communities) (line 6-7) Φ^*Q*^ from *C*^*k*^(*G*^*Q*^) (line 7-8).

## 4. Experiment and Result

We conduct our experiment on an academic coauthor (DBLP) dataset (Jie et al., 2008) and choose research papers that were published within 2005 to 2011. This revised dataset is a network of 15,516 authors with 48,862 co-author relationships between these authors and contains 193,512 research papers. The co-author information in DBLP is considered as interaction between the authors. We extract the authors' details, publication year, and abstract from each research paper. We apply latent dirichlet allocation (LDA) topic modeling (Blei et al., 2003) on the abstracts of the research papers in order to find the research topics.

**Comparison Methods**. We compare our Algorithm 1 (Query Algorithm), denoted here as TO-HIOC, with two other existing methods: HICD method (Lim and Datta, 2016) and iTop algorithm (Correa et al., 2012).

**Algorithm 1:** Query Algorithm

**Input:** *G*=(*U, E*), *Q, k*, θ
**Output:** set of active interactive communities Φ_*Q*_=$\left\{H_{1}^{k},H_{2}^{k},\ldots ,H_{n}^{k}\right\}$
1: for each (*u, v*)∈*E* **do**
2: compute *w*_*uv*_
3: if *w*_*uv*_>θ **then**
4: *U*^*Q*^.*add*(*u*)
5: *U*^*Q*^.*add*(*v*)
6: compute the induced graph *G*^*Q*^ on *U*^*Q*^
7: compute the maximal *k*-core *C*^*k*^(*G*^*Q*^) of *G*^*Q*^
8: Output the set of active connected components Φ_*Q*_ from *C*^*k*^(*G*^*Q*^)

**Evaluation Measures**. We vary the length of the *Q* to |*Q*| = 2, 3, 4 and use three measures of density, entropy and modularity to evaluate the quality of the detected online communities discovered by different methods. The definition of density, entropy, and modularity are as follows.

(2) $$\begin{array}{l} density\left({\left\{H_{j}^{k}\right\}}_{j=1}^{n}\right)=\overset{n}{\underset{j}{\sum }}\frac{\left|\left\{\left(u,v\right)|u,v\in H_{j}^{k},\left(u,v\right)\in E\right\}\right|}{\left|E\right|} \end{array}$$

where *n* denotes the total number of detected communities. Density measures the compactness of the communities in structure.

$$\begin{array}{lll} entropy\left({\left\{H_{j}^{k}\right\}}_{j=1}^{n}\right) & = & \overset{n}{\underset{j}{\sum }}\frac{\left|U\left(H_{j}^{k}\right)\right|}{\left|U\right|}entropy\left(H_{j}^{k}\right),\text{where } \end{array}$$

(3) $$\begin{array}{lll} entropy\left(H_{j}^{k}\right) & = & -\overset{n}{\underset{i=1}{\sum }}p_{ij}log_{2}p_{ij} \end{array}$$

and *p*_*ij*_ is the percentage of members in a community $G_{j}$ who are active on the query topic *T*_*i*_. $entropy\left({\left\{G_{j}\right\}}_{j=1}^{n}\right)$ measures the weighted entropy considering all the query topics over all the communities. Entropy indicates the randomness of the topics which are covered in the communities.

(4) $$\begin{array}{l} modularity\left({\left\{H_{j}^{k}\right\}}_{j=1}^{n}\right)=\frac{1}{2m}\underset{ij}{\sum }\left[A_{ij}-\frac{d_{i}d_{j}}{2m}\right]\delta \left(s_{i},s_{j}\right) \end{array}$$

Here, *m* denotes the number of edges corresponding to an adjacency matrix *A*^1^, *d*_*i*_ denotes the degree corresponding to node *n*_*i*_, *s*_*i*_ denotes the community membership of node *n*_*i*_ and δ(*s*_*i*_, *s*_*j*_) = 1 if *s*_*i*_=*s*_*j*_.

Generally, a good interactive community should have high density, high modularity, and low entropy.

**Figure 2:**
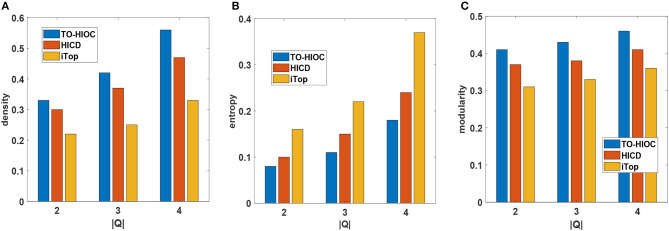
Performance comparison on DBLP dataset **(A)** Density, **(B)** Entropy, **(C)** Modularity (in all cases, *Q* = {Semantic web, Data mining, Social network analysis}, *k* = {4}, θ = {0.5}, γ = {4}, the publications are chosen from the time period of 2005 to 2009).

Figure 2A shows the density comparison between all the methods on the DBLP dataset. We set *k* = 4 as there are usually many small-sized research groups existing in DBLP. We see that TO-HIOC achieves better performance compared to the other two methods because it considers query-oriented active interactions among the community members. The HICD method fails to achieve better density values as it requires interaction between users (authors) to the celebrities (i.e., very high profile researchers in DBLP), which is not very common. The iTop method ignores the interactions between the non-seed users, resulting in poor performance. We also observe that all the methods achieve better density values for higher values of |*Q*|. The reason is that the number of interactive connections of the users increases as |*Q*|increases, which results in large and more densely connected communities.

Figure 2B shows the entropy comparison between the three methods. TO-HIOC achieves better performance in the aspect of the entropy as it considers the topical relevance (with regard to the query topics) during the interactions between the authors while forming a community. On the other hand, HICD achieves higher entropy value because not all the connected authors in a community have interests or active interactions in the common research topics. iTop also achieves a higher entropy value due to the lack of active topical interactions between the seed users and their followers. We see in Figure 2C that our proposed method TO-HIOC outperforms HICD and iTop in modularity comparison.

We examined a community in a co-author dataset which includes Jie Tang, who is one of the leading researchers in the data mining area, to see the differences in the community members for different values of *k* and *Q* = {semantic web, topic mining, social network analysis}.

**Figure 3:**
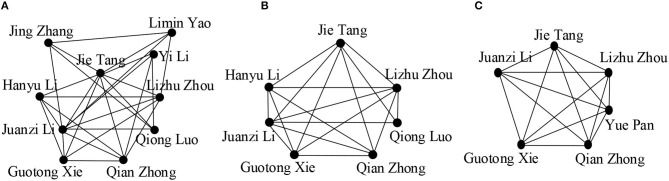
**(A)** *k* = {3}; **(B)** *k* = {4}; **(C)** *k* = {5}; (in all cases, *Q* = {Semantic web}, θ = {0.5}, γ = {4}, the publications are chosen from the time period of 2005 to 2009).

**Figure 4:**
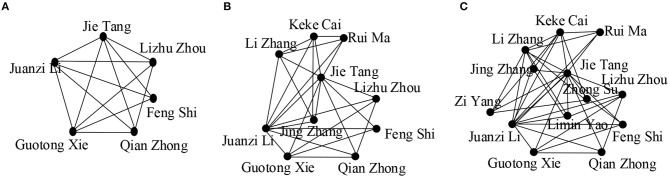
**(A)** *Q* = {Semantic web}; **(B)** *Q* = {Semantic web, Data mining}; **(C)** *Q* = {Semantic web, Data mining, Social network analysis}; (in all cases, *k* = {4}, θ = {0.5}, γ = {4}, the publications are chosen from the time period of 2007 to 2011).

We observe the effect of value *k* in Figures 3A–C. By varying the values of *k*, we get communities of different sizes. We see that the community size decreases for higher values of *k* as the cohesiveness constraint becomes more strict, resulting in the exclusion of some active community members, for example “Yi Li,” “Jing Zhang,” “Limin Yao”leave the group. We also see that more researchers joined the community when the length of *Q* is increased as higher values of |*Q*|covered more interactive researchers (Figures 4A–C).

## 5. Conclusion

In this paper, a topic-oriented highly interactive community detection approach is proposed. This method detects global communities where users have active interaction with each other on common topics. We observed that users have different degrees of interactions for different topics. As future work, we will consider the temporal factor to measure the recency behavior of users' interactions.

## Data Availability

The raw data supporting the conclusions of this manuscript will be made available by the authors, without undue reservation, to any qualified researcher.

## Author Contributions

For this research paper, SD conducted the experiments (100%), designed the algorithm (75%), and wrote the paper (70%). MA designed the algorithm and experiments (25%), revised the paper (30%) as well as provided helpful insights and contribution in problem formulation, idea refinement, reviewing, and polishing the paper.

### Conflict of Interest Statement

The authors declare that the research was conducted in the absence of any commercial or financial relationships that could be construed as a potential conflict of interest.

## Footnotes

^1^An adjacency matrix of a network is represented by *A*, where *A*_*uv*_ = 0 means there is no edge (no interaction) between nodes *u* and *v* and *A*_*uv*_ = 1 means there is an edge between the two.
